# Complete mitochondrial genome of the stingless bee *Lepidotrigona terminata* (Hymenoptera: Meliponinae) and phylogenetic analysis

**DOI:** 10.1080/23802359.2020.1715298

**Published:** 2020-01-21

**Authors:** Cheng-Ye Wang, Min Zhao, Huan-Li Xu, Feng-Long Zhang, Yi-Hai Zhong, Ying Feng, Shi-Jie Wang

**Affiliations:** a Research Institute of Resource Insects, Chinese Academy of Forestry, Kunming, China;; b Department of Entomology, College of Plant Protection, China Agricultural University, Beijing, China;; c Yunnan Fenghong Biotechnology Co., Ltd., Kunming, China;; d Environment and Plant Protection Institute, Chinese Academy of Tropical Agricultural Sciences, Haikou, China

**Keywords:** Mitogenome, *Lepidotrigona terminata*, stingless bee, phylogeny

## Abstract

*Lepidotrigona terminata* (Smith, 1878) is a stingless bee that distributed in Eastern Asia. The complete mitogenome of *L. terminata* (GenBank accession number MN737481) is 15,431 bp in size, including 13 protein-coding genes, 22 transfer RNAs, two ribosomal RNAs genes, and a noncoding D-loop region. The D-loop region is located between ND4L and tRNA^Met^, different from the other two stingless bee mitogenomes previously reported. The base composition of the whole *L. terminata* mitogenome is 38.18% for A, 11.67% for G, 38.32% for T, and 11.83% for C, with a high AT bias of 76.50%. The present data could contribute to a detailed phylogeographic analysis of this valuable economic insect for further study in differentiating closely related species.

*Lepidotrigona terminata* (Smith, 1878) (Hymenoptera: Meliponinae) is a species of stingless bee distributed in China (Wu 2000), Cambodia, Laos, Indonesia, Malaysia, Myanmar, Thailand, and Vietnam (Lee et al. 2016). As a pollinator, this stingless bee has great development value (Slaa et al. 2006). Elucidating the sequence and structure of *L. terminata* mitogenome is important for understanding its diversity and evolution.

The specimen of *L. terminata* was obtained from Puer, Yunnan, China (N23°32′, E100°50′) and deposited in the insect specimen room of Research Institute of Resource Insects with an accession number RIRI-w-20191012. Sequencing work of the complete mitogenome of *L. terminata* was performed using Illumina Nextseq500 in Beijing Microread Genetics Co., Ltd., with a total data volume 4 G (150 bp Reads). High-quality reads were assembled from scratch using IDBA-UD and SPAdes (Gurevich et al. 2013). Protein-coding genes (PCGs) of the *L. terminata* mitogenome were identified using BLAST search in NCBI, and tRNA genes were identified using the tRNAscan-SE search server (Schattner et al. 2005). The final assembled mitogenome was also verified on the MITOS web server (Bernt et al. 2013).

The gene order of *L. terminata* mitogenome is different from the other two stingless bee mitogenomes previously reported (GenBank accession numbers AF466146, KP202303), and several genes have been rearranged. The mitogenome is 15,431 bp in size (GenBank accession number MN737481), including 13 typical invertebrate PCGs, 22 transfer RNA genes, two ribosomal RNA genes and a noncoding control region (D-loop). The A + T content of the whole *L. terminata* mitogenome is 76.50%, showing an obvious AT mutation bias (Eyre-Walker 1997). The D-loop region exhibits the highest A + T content (86.42%) in the *L. terminata* mitogenome.

All 13 PCGs use standard ATN as a start codon. As for the stop codon, nine PCGs had the common mitochondrial stop codon TAA, whereas *COX1*, *ND3*, *ND1*, and *CYTB* terminated with stop codon TAG. All the tRNAs except *tRNA^Ser^ (UCU)* could be folded into the typical cloverleaf secondary structures. The unusual *tRNA^Ser^ (UCU)* lacks the dihydrouridine (DHU) arm.

Based on the concatenated 13 mitochondrial PCGs sequences of 18 species from Hymenoptera, the neighbour-joining method was used to construct the phylogenetic relationship of *L. terminata* with 17 other Hymenoptera insects (Figure 1). The phylogenetic analysis was performed using MEGA7 software (Kumar et al. 2016). *Lepidotrigona terminata* was clustered with the other two stingless bees (*Melipona scutellaris* and *Melipona bicolor*), and the phylogeny tree indicates that stingless bee has a closer relationship with bumblebees (*Bombus*) than with honeybees (*Apis*). This mitogenome data might be also useful for further phylogeography analyses in Meliponinae.

**Figure 1.**
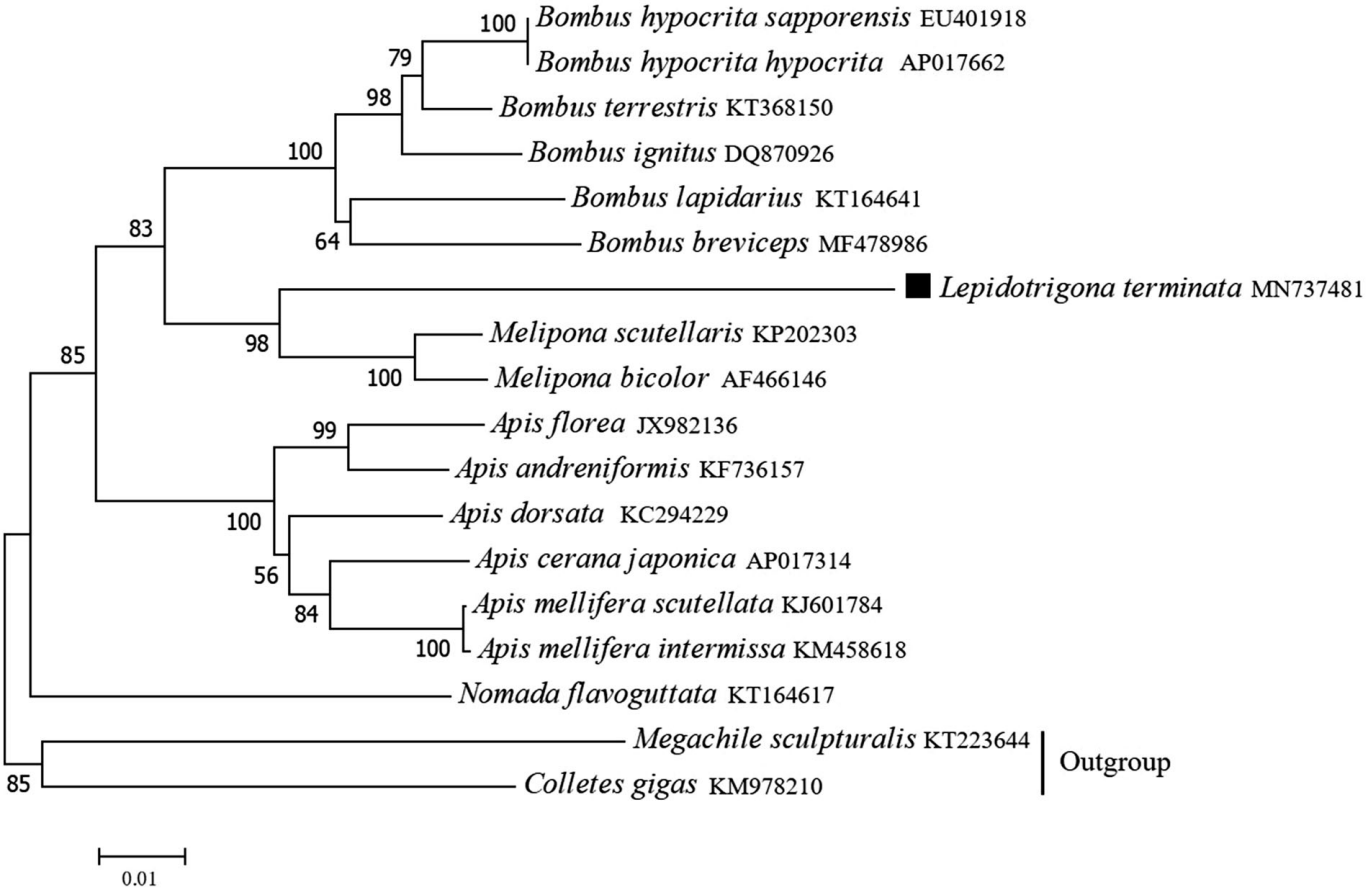
Phylogenetic tree showing the relationship between *L. terminata* and 17 other Hymenoptera insects based on neighbour-joining method. Megachilidae (*Megachile sculpturalis*) and Colletidae (*Colletes gigas*) were used as outgroup. GenBank accession numbers of each sequence were listed in the tree with their corresponding species names.
